# A Risk Assessment Framework Proposal Based on Bow-Tie Analysis for Medical Image Diagnosis Sharing within Telemedicine

**DOI:** 10.3390/s21072426

**Published:** 2021-04-01

**Authors:** Thiago Poleto, Maisa Mendonça Silva, Thárcylla Rebecca Negreiros Clemente, Ana Paula Henriques de Gusmão, Ana Paula de Barros Araújo, Ana Paula Cabral Seixas Costa

**Affiliations:** 1 Department of Business Administration, Federal University of Pará, Belém 66075-110, Brazil; 2 Department of Management Engineering, Universidade Federal de Pernambuco, Recife 50670-901, Brazil; maisa@cdsid.org.br (M.M.S.); anapaula.araujo@ufpe.br (A.P.d.B.A.); apcabral@cdsid.org.br (A.P.C.S.C.); 3 Department of Management Engineering CAA, Universidade Federal de Pernambuco, Caruaru 55002-970, Brazil; tharcylla.clemente@ufpe.br; 4 Department of Management Engineering, Universidade Federal de Sergipe, São Cristóvão 49100-000, Brazil; anapaulagusmao@cdsid.org.br

**Keywords:** image and diagnosis medical security, bow-tie analysis, cyberattack, cybersecurity, decision-making

## Abstract

The purpose of this paper is to propose a framework for cybersecurity risk management in telemedicine. The framework, which uses a bow-tie approach for medical image diagnosis sharing, allows the identification, analysis, and assessment of risks, considering the ISO/TS 13131:2014 recommendations. The bow-tie method combines fault tree analysis (FTA) and event tree analysis (ETA). The literature review supported the identification of the main causes and forms of control associated with cybersecurity risks in telemedicine. The main finding of this paper is that it is possible, through a structured model, to manage risks and avoid losses for everyone involved in the process of exchanging medical image information through telemedicine services. Through the framework, those responsible for the telemedicine services can identify potential risks in cybersecurity and act preventively, recognizing the causes even as, in a mitigating way, identifying viable controls and prioritizing investments. Despite the existence of many studies on cybersecurity, the paper provides theoretical contributions to studies on cybersecurity risks and features a new methodological approach, which incorporates both causes and consequences of the incident scenario.

## 1. Introduction

Information technology (IT) has been gaining wide use in the generation of information and in the decision support process in different contexts. One of the IT resources of greatest investment in recent years is the set of multimedia features that allows the sharing of texts, documents, sounds, and images in high resolution, being able to describe, reproduce, create, represent, and simulate several systems. In general, IT multimedia resources are indicated as facilitators of communication and content transmission, especially via the Internet and remote devices, and have ensured the availability and accessibility of different data formats for many organizational processes. As an example, it is possible to consider the widespread use of IT multimedia resources to support medical diagnoses and treatments promoted by health institutions worldwide [1,2,3].

Medical information contains specific and personal details about patients and their health status. In many situations, images are used as essential sources of information and play a fundamental role in the composition of medical diagnosis [4,5]. The storage of images in medical databases is widely recognized as a procedure that facilitates access to images by several doctors and health centers around the world, offering the sharing of information and knowledge about different health conditions [6]. In particular, via the Internet and distributed databases, patient reports can be downloaded quickly in order to streamline the response to the healthcare service and real-time monitoring using data from vision-based sensors [7].

The process of transferring medical images is included in the telemedicine resources that comprise several types of imaging tests used in medical diagnostics, such as computed tomography, magnetic resonance, radiography, mammography, nuclear medicine, and ultrasound [8,9]. One of the main storage environments for medical images, the picture archiving and communication system (PACS), allows images and results of treatments to be accessed and shared by several health centers around the world, which speeds up the development of exams, medications, and surgical techniques, in order to support research and discoveries that increase the life expectancy of the population, improve quality of life, and reduce mortality indicators [10,11]. However, in addition to images, personal and confidential data about patients are often shared on the network, which highlights the system’s vulnerability [12].

In general, the vulnerability of the system is related to the communication protocols and sharing of medical content via the Internet and distributed databases [13]. Researchers emphasize the concern regarding the implementation of techniques of data hiding, protection, and integrity of medical diagnosis, especially because this type of information is highly sensitive and, if corrupted or modified by cyberattacks, can lead to costly litigation and fines [14,15].

Despite the different techniques used by telemedicine to protect the information shared, cyberattacks can cause huge losses, leading to changes in diagnoses and an increased risk of spreading an incorrect report. Therefore, cybersecurity of medical data is critical in telemedicine solutions because the damage caused by cyberattacks can create serious problems in the diagnosis decision for any individual during the transfer of this data, given that cybersecurity can be considered as a non-cooperative game in which, on one hand, a hacker is trying to find vulnerabilities to exploit sensitive data or perform malicious actions, and on the other hand, the defender is continuously restricting the attack if a threat to the system occurs [16,17]. This process can be considered complex due to the lack of complete information about how or when a cyberattack will occur. However, it is opportune to develop analyses that may make it possible to understand the uncertain context.

The advancement of solutions in the cybersecurity area is driven by business growth in the digital age. Recent research has considered aspects of risk analysis to analyze the operational and strategic conduct of organizations in relation to the uncertainty of the impact of cyberattacks in organizational environments [18,19,20,21,22,23,24,25,26,27]. The study of the risks involved in cyberattacks is an opportunity to explore potential failure modes in the image and content archiving system, which can be considered motivators for investing in cybersecurity in telemedicine.

Therefore, the present work aims to address the problem related to cybersecurity within telemedicine with a focus on image and diagnosis sharing using a bow-tie approach. The contributions of this paper are threefold: (i) identification of potential risk factors and risk events; (ii) prioritization of actions that minimize the impact of cyberattacks on telemedicine resources; and (iii) recommendations for the construction of efficient security policies. To the best of our knowledge, this is the first paper that uses a qualitative–quantitative approach to perform a risk analysis regarding online images and online diagnosis within the telemedicine context. 

This paper is structured as follows. Section 2 presents a background on telehealth services and infrastructure, cyberattacks in telemedicine services, cybersecurity regarding medical images, and the bow-tie analysis. Section 3 is dedicated to the proposed framework for cybersecurity in telemedicine. Section 4 provides an illustrative example using the proposed framework. Finally, Section 5 shows the theoretical and practical implications of the paper, and Section 6 draws some conclusions. 

## 2. Background

### 2.1. Telehealth Service and Infrastructure

According to the American Telemedicine Association (ATA), telehealth can be understood as the natural evolution of healthcare in the current digital world because it uses telecommunications technologies and services to provide medical care. In this sense, telehealth can be defined as the use of a technology-based virtual platform to provide various forms of medical care and services at-a-distance, and telemedicine—telehealth’s largest segment—is the use of a remote electronic interface to provide the practice of medicine [28]. Therefore, a doctor in one location can use a telecommunication infrastructure to deliver care to a patient at a distant site.

The benefits of telehealth, as related by the ATA, include: (i) value creation for payers, patients, and providers (doctors and clinicians), since it is possible to manage more information about the health status of individuals; (ii) increased patient access to medical reports; (iii) enhanced reach of healthcare services because distance care can be an effective alternative; (iv) 24/7 coverage, which represents the total availability of healthcare services online; (v) higher customer satisfaction due to a high level of communication with the doctor and reduced waiting time for the diagnosis of illnesses; and (vi) reduced cost structure due to the growing offer of technological products and infrastructure that ensure data processing in virtual scales.

A telehealth service can be identified by the communication types and resources that support the telemedicine in different contexts. Table 1 shows the communication types, telemedicine tools, and services.

The functional requirement of the communication infrastructure that allows to obtain the benefits of telehealth is the proposal to integrate remote devices with electronic medical records [30]. The distribution of databases allows the storage and sharing of texts, documents, sounds, and medical images in high resolution, being able to describe, reproduce, create, represent, and simulate several diagnostics about potential diseases by doctors and clinical professionals worldwide [31]. 

Medical data can be structured, semi-structured or unstructured, or discrete or continuous. As a result, these features need to be considered in data analytics processing. In particular, data analytics processing in healthcare enables the analysis of large datasets from thousands of patients using various intelligent computational techniques to define standards that can assist in the development of medical reports and diagnostics, supporting resources of bioinformatics, medical imaging, medical informatics, and health informatics [32].

In recent years, experts have turned their attention to the growing risks and negative impacts that the lack of cybersecurity has caused in the evolution of business in cyberspace [33]. The lack of cybersecurity has negatively affected the trust of customers and suppliers, service efficiency, the availability of operations, the credibility of the business, and the image of the company. Considering the impact of these attacks, the cyberattacks related to medical services will be addressed in the next section. 

### 2.2. Cyberattacks in Telemedicine Services

The lack of training, in turn, is often due to the fact that managers have not well mapped the processes inherent to telemedicine services and the respective risks to privacy and the security of the inherent information [34]. Consider the situation where the patient’s medical information is transferred from one doctor to another doctor for a better solution and health treatment and classification of android malware images [35]. Several possibilities of threats, provided by the transmission of data through a communication channel, can severely affect their authenticity, integrity, and confidentiality [36]. 

**Figure 1:**
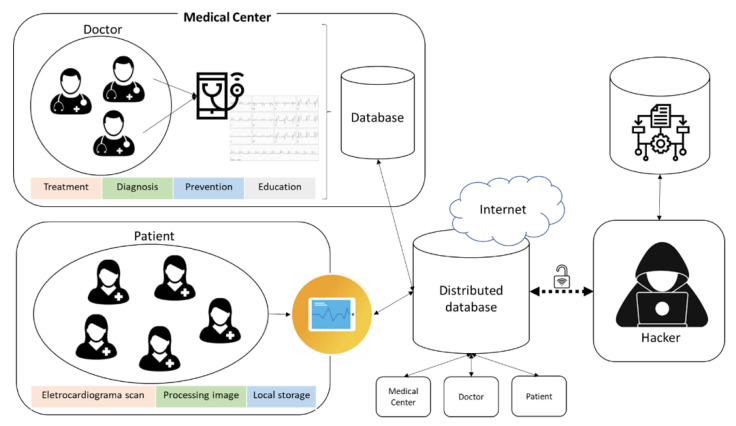
Cyberattacks in telemedicine services.

Clinical professionals face a challenge in protecting the privacy of patients in the process of transferring the medical image diagnosis. Information management in telemedicine is a domain that requires proactive actions using techniques such as cryptography, digital signature, and anonymity. However, the scenario can be even more complex when it comes to cyberattacks. Some of these failures can be accidental, or they can be a general neglect to guarantee confidential information in telemedicine services. Figure 1 shows a cyberattack related to the communication using IT devices among clinical professionals in medical centers and patients. 

Effective communication in telemedicine services is supported by the infrastructure of distributed databases that, via the Internet, allows the sharing of data, texts, documents, sounds, and images. However, despite the benefits that this structure offers, there are vulnerabilities that can threaten the integrity of the stored data and cause enormous harm to patients. According to [37], four types of attacks can occur during communication established in telemedicine services: interruption, interception, modification, and fabrication.

In general, vulnerabilities are inserted into the system due to inefficiency or lack of adequate information security policies. Such vulnerabilities allow threats such as hacker actions to manipulate, steal, remove, disable, interrupt, or corrupt data, texts, documents, sounds, and images of the distributed database, causing large-scale damage and compromising the evolution of the business. Hackers, as shown in Figure 1, consider the organization’s behavior, as well as vulnerable services and applications to create convincing e-mail messages to entice users to open an attachment, to visit an infected website, or to disclose security credentials in response to a contrived message. These actions are frequent attack mechanisms that have been proven to be very successful.

The risk, which is the probability that a threat exploits a certain vulnerability in a system, is associated with the manifestation of the threat [18,38,39]. Often, the risk identification is not completely known due to a lack of knowledge about when or how the threat will manifest itself in the system. For example, there are no precise standards for identifying when a hacker will act and corrupt images in a medical database. However, it is possible to classify risks by the different causes of losses or their impacts or consequences in a given system. Particularly, in telemedicine services, there are concerns about the integrity of medical images and diagnoses. 

It is worth stating that the analysis of cybersecurity risk regarding medical images is highlighted by normative documents to IT managers in reducing or eliminating adverse events in telemedicine services. Therefore, the next subsection is dedicated to the topic of cybersecurity risk regarding medical images including information about norms and techniques in which cybersecurity in telemedicine can be based on to minimize risks. 

### 2.3. Cybersecurity Risk Regarding Medical Images

Despite the advantages conferred by telehealth, attention must be paid to information security issues. When healthcare practices are performed using the Internet and all information is electronic, ensuring the security and privacy of clinical information becomes more complex. As previously mentioned, this is partly because most health professionals are not trained in protecting the security and privacy of patients in cyberspace. To make things worse, there are many methods that can be used to break into the electronic system and gain unauthorized access to a large amount of health information.

Due to the negative impact that cyberattacks can have on telemedicine services, there are some methods and techniques to ensure the protection of medical images, including discrete wavelet transform (DWT) [40,41,42] chaos system (CS) [43,44] zero watermarking [45] SDGOEF (Shearlets and DRPE-based generalized optical encryption framework) [46], support vector machine (SVM) [47,48], fuzzy C-means clustering (FCM) [26,48]; Internet of Medical Things–security assessment framework (IoMT-SAF) [49], fuzzy chaotic maps [42], neural network (NN) [41,50], RSA encryption [41]; quantum walks [51], and Multiple Image Owners with Privacy Protection (MIPP) [52].

In recent years, various safety parameters and standards have been developed by agencies, institutes, and researchers to protect medical information in telemedicine services [53]. For instance, in 2008, the first safety standard for medical images, known as ISO 27799:2008, was created by the International Organization for Standardization (ISO). Although the ISO rules provide a standard for regulating the collection and dissemination of health information, several countries have developed their own safety standards for medical imaging. For example, in the USA, in addition to the use of ISO standards, the Health Insurance Portability and Accountability Act (HIPAA) was created to provide privacy and security rules and regulations to protect PHI (protected health information) available to insurance providers as properly governed. The European Union (EU) launched the GDPR (General Data Protection Regulation), which protects all personal data belonging to users residing in the EU and meets the challenges of personal health data protection.

With a focus on risk management in telemedicine service, ISO/TS 13131:2014 [54] stands out for being the most used standard. This norm provides advice and recommendations on how to develop quality objectives and guidelines for telehealth services that use information and communications technologies (ICTs) to deliver healthcare over both long and short distances by using a risk management process. Due to these factors, ISO/TS 13131:2014 will be used as a reference for the framework proposed herein. 

Finally, the main control measures adopted to provide security to medical images in telemedicine are watermarking, digital fingerprinting, encryption, and digital signature algorithm. The adoption of these techniques is associated with the main objectives of ensuring the security of medical information. In this sense, Ref. [55] argue that in the process of ensuring information security in telemedicine services, three characteristics related to the types of attacks covered in Section 2.2 are mandatory: confidentiality; reliability which addresses integrity and authentication; availability.

Besides the technical aspects of medical image cybersecurity, it is worth stating the importance of using a risk assessment method to deal with cybersecurity within telemedicine. Thus, the bow-tie analysis is presented in the next section along with the reasons why this methodology is suitable for the telemedicine context. 

### 2.4. Bow-Tie Analysis

Many risk assessment methods including qualitative and quantitative techniques, such as checklists, hazard and operability study (HAZOP), fault tree analysis (FTA), event tree analysis (ETA), failure mode and effect analysis (FMEA), and hazard indices, have been designed for risk assessment in several contexts. In a general way, the bow-tie approach was created for security management and used mainly to identify threats, analyze barriers, and assess operational risks. In this sense, bow-tie analysis, which is a combination of FTA and ETA, is very popular because it incorporates both the causes and consequences of the incident scenario and can be used to assess all kinds of risks such as the ones regarding gas and oil pipelines [56,57], occupational risks [58], and industrial risks [59,60].

**Figure 2:**
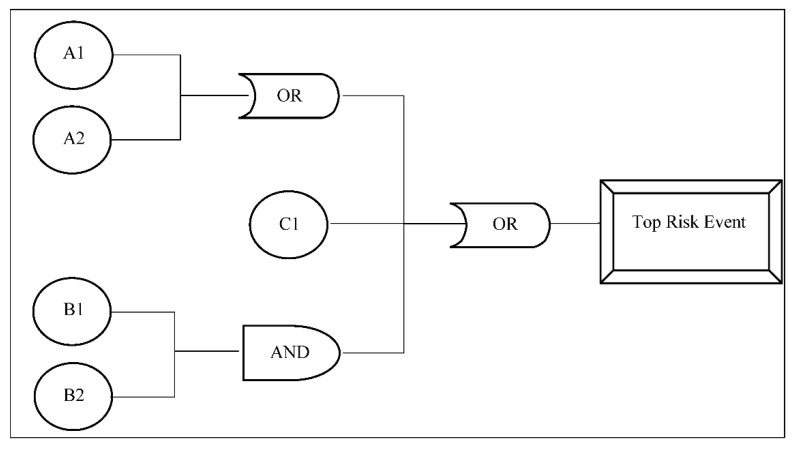
General fault tree.

**Figure 3:**
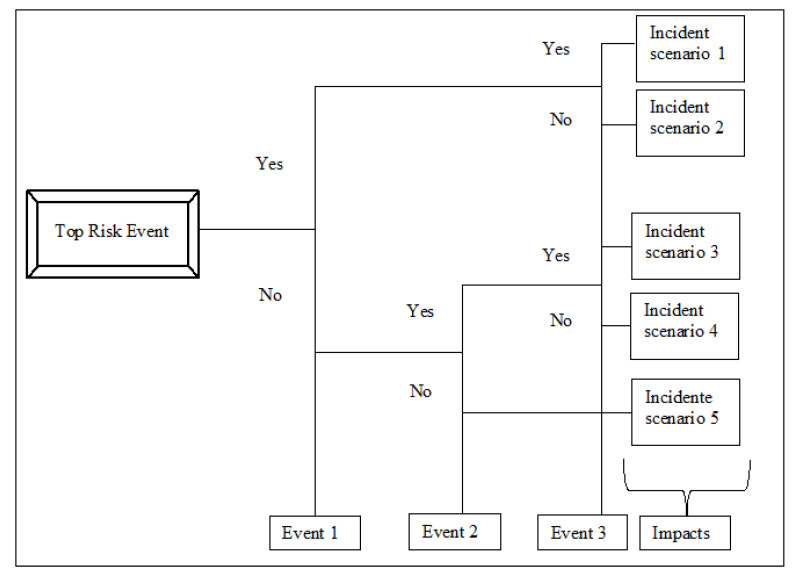
General event tree.

The bow-tie diagram is designed in a way that the fault tree (FT) is placed on the left side and the event tree (ET) is placed on the right side. On the one hand, the FT starts with the critical event (top risk event) [61] and goes to the left side until the intermediate causes are described in terms of basic events with the use of logical connections of type “AND” and “OR”. On the other hand, the ET starts with the same critical event (top risk event) and follows the sequence of events to the right side using “AND” connections until it reaches the final outcome events. Figure 2 shows a general FT, and Figure 3 shows a general ET.

With regard to an FT, the output of an *OR* gate occurs if some input occurs, and the output of an *AND* gate occurs if all inputs occur. Therefore, given the estimated probabilities of occurrence for risk factors and using the FT of the corresponding bow-tie diagram, the probability of occurrence for the risk event can be calculated assuming that the risk factors are independent. This is the default assumption of a traditional bow-tie analysis. However, several approaches can be used to assume that there are relationships among failure events such as correlation [56] and conditional probability [60]. Thus, the probabilities of an *OR* gate and an *AND* gate are calculated, respectively, as:

(1) $$P_{OR}=\overset{n}{\underset{i=1}{\sum }}p_{i}\;,$$

(2) $$P_{AND}=\;\overset{n}{\underset{i=1}{\prod }}p_{i},$$

where i is a specific basic event, n is the total number of basic events which generate the risk event under analysis, and $p_{i}$ is the probability of occurrence of the basic event i. For instance, the probability of occurrence of the top risk event in Figure 2 is:

(3) $$P_{RE}=(P_{A_{1}}\times \;P_{A_{2}})\;+\;P_{C_{1}}\;+\;(P_{B_{1}}\;\times \;P_{B_{2}}).$$

With regard to an ET, different incident scenarios may result from a combination of events following a top risk event. Moreover, the ET considers binary situations such as “Yes” and “No” to propagate the intermediate events until all possible output events are represented in the bow-tie diagram. The severity of the outcomes can be calculated according to the impacts that they can generate. Normally, the impacts are regarding economic, environmental, social, and political aspects (among others). 

## 3. Proposed Framework for Cybersecurity in Telemedicine

**Figure 4:**
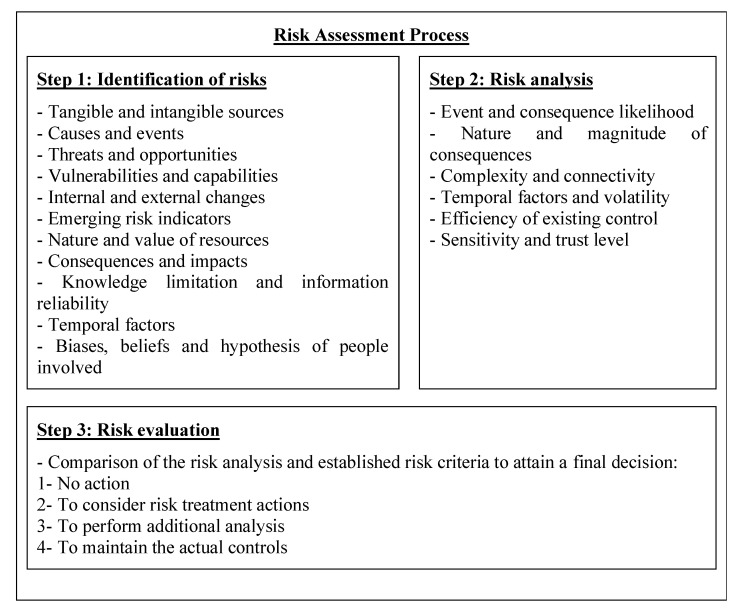
Three steps of the risk assessment process (Source: ISO/TS 13131:2014).

According to [54], which provides advice and recommendations on how to develop quality objectives and guidelines for telehealth services that use ICTs, and based on ISO 31100:2018, which provides general guidelines for risk management, the risk management process involves: (i) the systematic application of policies, procedures, and practices for the communication and inquiry activities related to risks; (ii) the establishment of context and evaluation of risks; and (iii) the treatment, monitoring, critical analysis, recording and reporting of risks. Moreover, with a focus on (ii), the risk assessment process comprises the identification of risks, risk analysis, and risk evaluation as shown in Figure 4.

**Figure 5:**
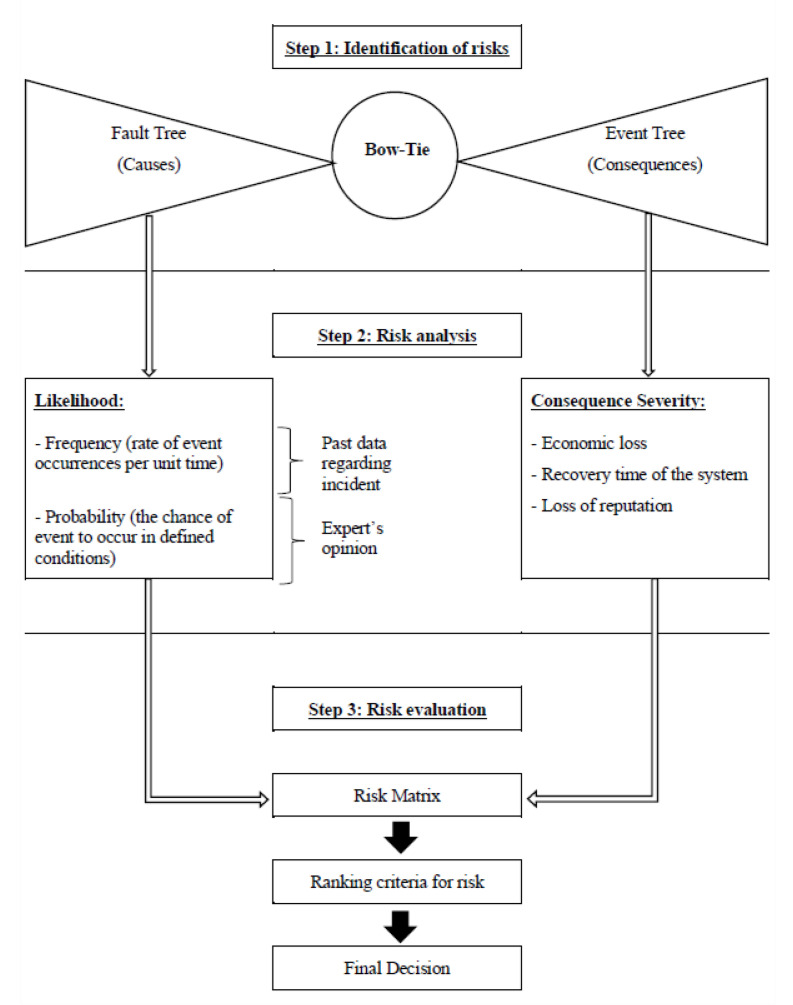
Proposed framework for cybersecurity in telemedicine.

Following the steps of the risk assessment process proposed in ISO/TS 13131:2014, the framework for cybersecurity in telemedicine proposed in this paper comprising three steps is shown in Figure 5.

In Step 1, the identification of risk is conducted using a bow-tie analysis. This step is very critical because it is responsible for the identification of causes (which can be of three types: primary, intermediate, and top), preventive barriers, mitigating barriers, consequences, and the connections among them. In Step 2, the risk analysis is performed using likelihood of causes and severity of consequences. The likelihood of causes can be obtained by using past data regarding incident or expert knowledge. Finally, in Step 3, the risk evaluation is made integrating the risk analysis of Step 2 into a risk matrix where the two axes are: x—severity of consequences and y—probabilities of causes. Then, the established ranking criteria for risk are used to recommend a final decision which can be: (i) to take no action; (ii) to consider risk treatment actions; (iii) to perform additional analysis; and (iv) to maintain actual controls. The next section presents an illustrative example of how the proposed framework can be used for risk management in telemedicine.

## 4. Results and Discussion

The idea of this section is to show how the proposed model can assist managers in identifying risks to information security when providing health services. Based on the framework proposed (Figure 5), managers will be able to develop preventive and mitigating actions to be implemented by numerous health professionals. Therefore, a bow-tie analysis for cybersecurity in telemedicine is performed based on Figure 5, as follows.

### 4.1. Step 1: Identifications of Risks 

**Figure 6:**
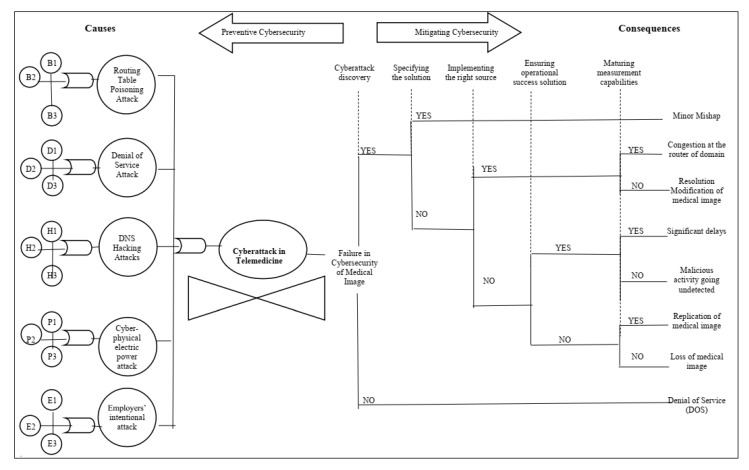
Bow-tie risk analysis for cybersecurity in telemedicine.

Cyberattacks can cause serious consequences such as information dissemination, falsification, service failure, server congestion, and changes in medical image resolution. In this way, the bow-tie methodology contributes to improve cybersecurity practices in telemedicine, which is affected by several factors including training rate, security policies, security certification, risk management capacity, IT governance, and management security costs [60]. The bow-tie model for cybersecurity in telemedicine is based on the related literature (which are shown in Table 2 and Table 3). The main critical elements in telemedicine cybersecurity can be divided according to Figure 6: causes (and/or gates, basic and intermediate failure events) consequences (events and incident scenarios), preventive cybersecurity, and mitigating cybersecurity.

Traditional information security assessments provide only a simple analysis of a critical event and do not effectively reveal the causes and consequences necessary to improve the transfer of medical images. Therefore, selecting appropriate cybersecurity control actions for telemedicine is an important task that requires a fundamental understanding of the organization’s business priorities. This understanding can demonstrate how to ensure more confidentiality, integrity, and availability of information effectively.

In this work, we analyze a critical event—a cyberattack in telemedicine—using the bow-tie methodology, to identify: (i) the causes (to act preventively) and (ii) the consequences (to provide a correction plan) of telemedicine cybersecurity. First, preventive cybersecurity (left part of Figure 6) can be performed by outlining the main causes of a cyberattack in telemedicine and the failure events from which they originate. This information is shown in Table 2 along with literature references and the index used in Figure 6. 

Second, organizations that adopt telemedicine practices must adequately mitigate the risks arising from the use of IT in the execution of their business functions while keeping their commitment to patients. Thus, mitigating cybersecurity (the right part of Figure 6) can also be done to provide a continuity plan for the health provider. In this way, the control analysis prescribes actions related to cybersecurity to be performed in telemedicine. The specification provides safety capability instructions to: (i) add control functionality/specificity; and/or (ii) increase the control force, due to possible adverse organizational impacts based on organizational risk assessments (Table 3). These instructions include both preventive and mitigating barriers. 

### 4.2. Step 2: Risk Analysis 

Based on Figure 6 and in possession of available data regarding likelihood of failures and severity of consequences, a risk analysis can be performed. First, as shown in Figure 5, the likelihood of failures can be obtained by two ways: past data regarding past failure events or expert knowledge. The probability of each intermediate failure cause can be calculated by using Equations (2) and (3). Table 4 shows these probabilities. 

Second, the severity of consequences can be estimated according to some criteria such as economic loss (in monetary units), recovery time of the system (in time units), and loss of reputation (number of dissatisfied patients). For instance, the impact of each incident scenario according to each criterion can be calculated by the expected value, which is the product of the probability of each event and the estimated loss in the corresponding units. The aggregation of each impact into a unique measure can be done as in [19]. Table 5 presents how the severity of consequences of incident scenarios can be calculated. 

### 4.3. Step 3: Risk Evaluation

**Figure 7:**
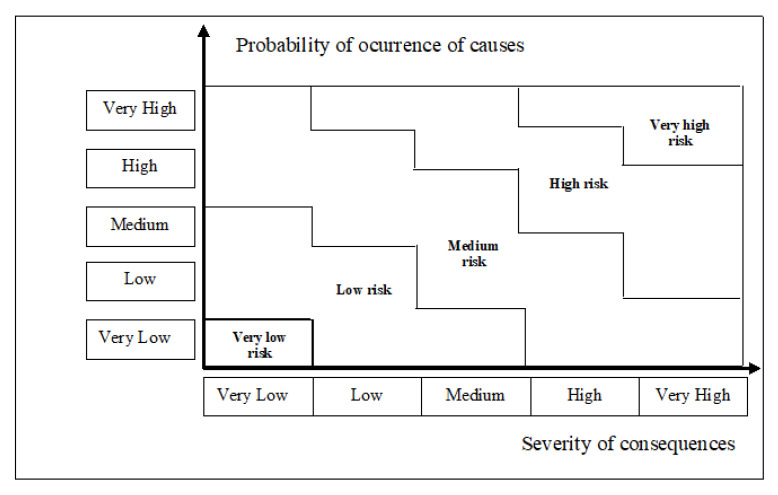
A 5 × 5 risk matrix for cybersecurity in telemedicine.

After the calculation of the probability of causes and severity of consequences, a risk matrix can be obtained (Figure 7), and the risks can be classified into levels ranging from very low to very high. For instance, as can be seen in Figure 7, risks can be classified as being low if the probability of occurrence of causes are low or medium and the severity of consequences are low or medium. Finally, a final decision can be recommended as a result of risk evaluation (Figure 4). 

## 5. Theoretical and Practical Implications

This paper has both theoretical implications for the cybersecurity literature and practical implications for IT managers. First, the existing cybersecurity literature mainly focuses on: (i) individual solutions to improve the automatic detection system [21,22,61]; (ii) presentation of cybersecurity architecture and standards [23,62,63], and (iii) development of algorithms for medical image encryption [64,65,66]. Therefore, despite the existence of many studies on cybersecurity, this study provides theoretical contributions to cybersecurity literature because it is the first paper that incorporates a qualitative–quantitative methodological approach designed for medical image security.

The causes related to the problems of routing table poisoning attacks, denial of service attacks, DNS haching attacks, cyber-phsical eletric power, employer’s intentional attacks. These factors, if not controlled, generate increased costs. The study by [18] identified some causes related to human error. They justified, in a function of current attacks that are more exposed to risks and uncertainties due to complexity in planning and design. Barriers were also established in telemedicine, in order to neutralize or minimize it. The main barriers were directed to investment in technology and operations monitoring actions. The use of performance indicators allows to verify how the process is behaving and, therefore, allows its flaws to be identified.

The remaining suggestions audit, security assessment, access control, provide cybersecurity advancement in telemedicine, such as the use of software. These solutions may not be low-cost or are complicated to implement but are worth investigating further to enhance the current controls in place for the hazards considered.

Second, with regard to the practical implications, the present study presents a framework with an integrated view to identify potential risks in cybersecurity related to the provision of telemedicine services that can be implemented in practice and that at the same time helps IT managers in clarifying the role of cybersecurity actions in real risk situations. 

Given this context, safety guidelines help the flow of reports and improve efficient communication, consequently enhancing IT risk management and the efficiency of the medical center in carrying out more accurate diagnoses. More precisely, the results from this study generated some insights that allow IT managers to improve cybersecurity policies in order to provide privacy guidelines to help organizations ensure confidentiality, availability, and integrity in telemedicine services. 

Another important point is that, currently, organizations do not always adopt defined and regulated cybersecurity standards, which can result in negative consequences for the remote healthcare system. In this sense, this study may help organizations in establishing a systematic way to perform cybersecurity within telemedicine. Thus, it is yet another contribution to the healthcare industry. 

Finally, this study can also be valuable for patients when it helps to clarify important aspects to ensure the privacy of personal data by promoting value in the provision of remote medical care and encouraging patients to become users of telemedicine services. The next section draws some conclusions and presents limitations and future works. 

## 6. Conclusions, and Future Works

In general, identifying effective actions for operational cybersecurity prevention is a challenge for organizations that design, implement, and operate complex information systems with several integrated hardware and software components. In many cases, cyberattacks occur due to the lack of adequate information security policies and the lack of understanding of IT resources by users. 

In the context of healthcare services, telemedicine solutions are, by nature, an integration of different parts and environments using remote devices and the Internet, to maintain efficient communication between the agents involved in the process of distance healthcare. Thus, there is a concern that IT managers must be able to understand the threats and risks during the process of developing cybersecurity policies to ensure the protection of patients’ personal data. From this perspective, the most relevant contribution of this paper is the identification of causes, consequences, and preventive and mitigating measures for threats that in some situations are neglected due to the complexity of a cybersecurity system in telemedicine services.

In particular, although the transmission of medical images online is arousing great interest, the images are vulnerable when they are stored in hospital storage or when they are transferred over an open transmission medium in various telemedicine applications. Cyberattacks are a problem that affects the health sector and can be life-threatening due to vulnerabilities in the telemedicine system. So, when implementing risk assessment measures in cybersecurity, it is possible to design an IT service level that ensures the security and privacy of information and maintains the reputation of healthcare organizations. 

Therefore, our proposed framework focuses on a multidimensional view of prevention to be applied to the context of telemedicine and consists of a set of policies and procedures to implement cybersecurity controls that prevent breaches of privacy and misuse or malicious use. It also emphasizes the importance of understanding cyberattack threats and allows structured visualization to help IT managers to better plan and improve the security of telemedicine systems regarding vulnerabilities. More particularly, the bow-tie approach was applied to identify the most critical causes of cyberattack scenarios and to quantify their consequences regarding medical image sharing.

Hospital companies, do not invest in cybersecurity training their staff to train their employees in all necessary processes, especially when there is a high turnover of manual labor, but also in the management. In general, these professionals lack adequate training during the work, but they have little or no knowledge about the physical, and characteristics of each type of software. In addition, investment in cybersecurity training is fundamental to change general behavior in changing organizational culture.

Therefore, we suggest that the main obstacles to implementing cybersecurity in telemedicine are: lack of planning for a cybersecurity project; lack of compliance with pre-established technical standards; choosing inappropriate software; lack of technical knowledge of the physical telemedicine about operations and services; lack of incentives and regulations aimed at cybersecurity

The proposed structure encourages the use of historical data or the knowledge of experts to carry out an analysis on the occurrence of risks and to indicate the relationship between the probability of causes and the severity of the consequences of cyberattacks. Thus, the risk matrix is integrated to provide the classification of the estimated risk and indicate the best recommendation for the decision on mitigating threats to telemedicine services. The applicability of the new framework proposal was done by means of an illustrative example. The main suggestion for future research is real-time implementation of data hiding techniques using new watermark techniques based on machine learning algorithms. 

## Figures and Tables

**Table 1:** Communication types in telemedicine services [29].

Communication Types	Telemedicine Tools	Telemedicine Services
Doctor to Doctor or Medical Center	E-mail and/or video	Dermatology, radiology, surgical peer mentoring, emergency trauma, and ICU care
Doctors to Patient	Video, phone, e-mail, remote wireless monitoring, Internet	Care for chronic conditions, medication management, wound care, counseling, post-discharge follow-up, mental health
Patient to Mobile Health Technology	Wearable monitors, smartphones, mobile apps, video, e-mail, web portals, games	Health education, monitoring of physical activity, monitoring of diet, medication adherence, cognitive fitness

**Table 2:** Failure events of cyberattacks in telemedicine.

Causes	References	Index	Failure Events of Cyberattacks in Telemedicine
Routing Table Poisoning Attacks	[24,59]	B1	Lack of node authentication
		B2	Updating routing table
		B3	Lack of verifying peers in index table
Denial of Service Attack	[60,61]	D1	Smurf attack
		D2	SYN flood
		D3	Botnets
DNS Hacking Attacks	[62,63]	H1	Cybersquatting
		H2	Human attacks
		H3	Authentication vulnerability
Cyber-Physical Electric Power	[61,64]	P1	Inadequate periodic security audits
		P2	Inadequate incident response process
		P3	Insufficient redundancy
Employers’ Intentional Attacks	[17,19,23]	E1	Insufficient trained personnel
		E2	Inadequate security awareness program
		E3	Third party as an agent of the utility having access to patient

**Table 3:** Preventive and mitigating cybersecurity.

Category	References	Preventive and Mitigating Cybersecurity	**Description**
Access Control	[61,62,63,64,65]	Notification of System Use	Granting access to the system that provides privacy and consistent security notices.
		Previous Logon (Access) Notification	Applicable to logons to information systems via human user interfaces.
		Session Termination	System automatically terminates a user session
		Remote Access	Establishes usage restrictions, configuration/connection requirement, privileged commands, monitoring for unauthorized connections, disable access.
Awareness and Training	[32,33]	Security Awareness Training	Provides basic security awareness training to information system users.
		Role-Based Security Training	Provides role-based security training to personnel with assigned security roles and responsibilities.
Audit and Accountability	[32,33,34,35,36,37]	Audit Events, Review, Analysis, and Reporting	Generates audit records containing information that establishes what type of event occurred, when the event occurred, and where the event occurred.
		Monitoring for Information Disclosure	Organization monitors evidence of unauthorized disclosure of organizational information.
Security Assessment and Authorization	[35]	System Interconnections	Control applies to dedicated connections between information systems and does not apply to transitory, user-controlled connections such as e-mail and website browsing.
		Security Authorization	Management decisions, conveyed through authorization decision documents.
		Continuous Monitoring	Programs facilitate ongoing awareness of threats, vulnerabilities, and information security to support organizational risk management decisions.
		Penetration Testing	Specialized type of assessment conducted on information systems or individual system components to identify vulnerabilities that could be exploited by adversaries.
Configuration Management	[21,65]	Information System Component Inventory	Control includes changes to baseline configurations for components and configuration items of information systems, changes to configuration settings for information technology products (e.g., operating systems, applications, firewalls, routers, and mobile devices), unscheduled/unauthorized changes, and changes to remediate vulnerabilities.
		Software Usage Restrictions	Provided under software license agreements that permit individuals to study, change, and improve the software.
		Security Impact Analysis	Organization analyzes changes to the information system to determine potential security impacts prior to change implementation.
Identification and Authentication	[61,62]	Device Identification and Authentication	Organizational devices requiring unique device-to-device identification and authentication may be defined by type, by device, or by a combination of type/device.
		Service Identification and Authentication	Architectural approaches requiring the identification and authentication of information system services.
		Cryptographic Module Authentication	Information system implements mechanisms for authentication to a cryptographic module.
Physical and Environmental Protection	[48,60]	Physical Access Authorizations	Control applies to organizational employees and visitors; individuals (e.g., employees, contractors, and others) with permanent physical access authorization credentials are not considered visitors.
		Fire Protection	Fire suppression and detection devices/systems for the information system that are supported by an independent energy source.
		Emergency Power	Provides a short-term uninterruptible power supply to facilitate in the event of a primary power source loss.
		Temperature and Humidity Controls	Control applies primarily to facilities containing concentrations of information system resources, for example, data centers, server rooms, and mainframe computer rooms
System and Communications Protection	[16]	Trusted Path	Information system establishes a trusted communications path between the user and the following security functions of the system.
		Cryptographic Protection	Establishes and manages cryptographic keys for required cryptography employed within the information system.
		Mobile Code	Information systems are based on the potential for the code to cause damage to the systems if used maliciously.

**Table 4:** Probability of causes that lead to cyberattacks in telemedicine.

Intermediate Causes	Index	Basic Failure Events	Gate Type	Probability
Routing Table Poisoning Attacks	B1	Lack of node authentication	*OR*	$P_{B1}+P_{B2}+P_{B3}$
	B2	Updating routing table		
	B3	Lack of verifying peers in index table		
Denial of Service Attack	D1	Smurf attack	*OR*	$P_{D1}+P_{D2}+P_{D3}$
	D2	SYN flood		
	D3	Botnets		
DNS Hacking Attacks	H1	Cybersquatting	*OR*	$P_{H1}+P_{H2}+P_{H3}$
	H2	Human attacks		
	H3	Authentication vulnerability		
Cyber-Physical Electric Power	P1	Inadequate periodic security audits	*OR*	$P_{P1}+P_{P2}+P_{P3}$
	P2	Inadequate incident response process		
	P3	Insufficient redundancy		
Employers’ Intentional Attacks	E1	Insufficient trained personnel	*OR*	$P_{E1}+P_{E2}+P_{E3}$
	E2	Inadequate security awareness program		
	E3	Third party as an agent of the utility having access to patient		

**Table 5:** Failure events of cyberattacks in telemedicine.

Incident Scenarios	Incident Events	Gate Type
Minor mishap	Cyberattack discovery Specification of a solution	*AND*
Congestion at the router of domain	Cyberattack discovery Implementation of the right source Maturation of measurement capabilities	*AND*
Resolution modification of medical image	Cyberattack discovery Implementation of the right source	*AND*
Significant delays	Cyberattack discovery Ensuring operational success solution Maturation of measurement capabilities	*AND*
Malicious activity undetected	Cyberattack discovery Ensuring operational success solution	*AND*
Replication of medical image	Cyberattack discovery Maturation of measurement capabilities	*AND*
Loss of medical image	Cyberattack discovery	*AND*
Denial of service (DoS)	Failure in cybersecurity of medical image	*AND*

## Data Availability

The data presented in this study are available on request from the corresponding author.
